# The Role of Antiphospholipid Antibodies in COVID-19

**DOI:** 10.1007/s11926-021-01041-7

**Published:** 2021-07-14

**Authors:** Maximilian Stelzer, Jörg Henes, Sebastian Saur

**Affiliations:** 1 School of Medicine, University of Tuebingen, Tuebingen, Germany; 2 Centre for Interdisciplinary Clinical Immunology, Rheumatology and Auto-inflammatory Diseases and Department of Internal Medicine II (Oncology, Hematology, Immunology, Rheumatology, Pulmology), University Hospital Tuebingen, Otfried-Mueller-Str. 10, DE 72076 Tuebingen, Germany

**Keywords:** Antiphopsholipd antibody, Antiphospholipid syndrom, Covid-19

## Abstract

**Purpose of the Review:**

Elevated levels of anti-phospholipid (aPL) antibodies are the most important criterion in the diagnosis of anti-phospholipid syndrome (APS) and are usually responsible for promoting the risk of thrombotic complications. Now, in the course of the global coronavirus disease 2019 (COVID-19) pandemic, measurable aPL antibodies have also been detected in a noticeable number of patients showing a variety ranging from studies with only isolated positive tests to cohorts with very high positivity. Thus, the question arises as to whether these two different clinical pictures may be linked.

**Recent Findings:**

The ambivalent results showed a frequent occurrence of the investigated aPL antibodies in COVID-19 patients to an individually varying degree. While some question a substantial correlation according to their results, a number of studies raise questions about the significance of a correlation of aPL antibodies in COVID-19 patients. Within the scope of this review, these have now been described and compared with each other.

**Summary:**

Ultimately, it is necessary to conduct further studies that specifically test aPL antibodies in a larger context in order to make subsequent important statements about the role of APS in COVID-19 and to further strengthen the significance of the described comparisons.

## Introduction

COVID-19 is an infectious disease, which can occur as a result of an infection with the novel severe acute respiratory syndrome coronavirus 2 (SARS-CoV-2). While a large proportion of COVID-19 patients remain in the clinical state of a viral respiratory infection, in some a development into a much more drastic and systematic illness leading to severe lung damage, multiple organ failure, and coagulopathy can be observed [1].

The COVID-19-associated coagulopathy (CAC) is of particular interest due to the fact that it could represent a new type of coagulopathy, which has many points overlapping with sepsis-induced coagulopathy (SIC), disseminated intravascular coagulation (DIC), hemophagocytic syndrome (HPS), thrombotic microangiopathy (TMA), and APS being of particular relevance for the subsequent analysis of APS in COVID-19, but in addition, showing numerous characteristics of its own [2]. Especially in patients treated in intensive care units (ICUs), a high incidence of thrombotic complications, manifesting mainly as pulmonary embolisms, can be assumed as seen in the exemplary figure of 31% during a series of 184 patients diagnosed with COVID-19 pneumonia in three Dutch hospitals [3].

On the contrary, APS is an autoimmune disease characterized by the formation of aPL antibodies such as anti-cardiolipin (aCL), anti-β2 glycoprotein I (anti-β2GPI), and lupus anticoagulant (LAC), leading to thrombophilia [4]. Clinically, APS often manifests itself in thromboembolism, miscarriages, or diseases during pregnancy and further non-criteria symptoms associated with APS such as thrombocytopenia and pulmonary hemorrhage [4, 5]. The prevalence figures of aPL antibodies involving a healthy population are found in studies with relatively low percentages; e.g., in a healthy control cohort of 200 people, IgG/IgM/IgA aCL 1%/1%/3%, and IgG/IgM/IgA anti-β2GPI 4%/1%/1% showed elevated levels [6].

In another review, which also reported about antibody studies of a healthy population, these were observed to be in a range from 1 to 5.6% [7].

Furthermore, the diagnostic criteria for the confirmation of APS should be mentioned, which correspond to the revised Sapporo criteria. Thus, it is necessary that one clinical and one laboratory criterion are met; this requirement and its problems are discussed in more detail in the “Discussion” [8].

Interestingly, infections may even be a possible trigger for the occurrence of APS, stimulating the production of APL antibodies, i.e., through molecular mimicry, viral examples of which possibly include human immunodeficiency virus, hepatitis B virus, and hepatitis C virus [4, 9].

Since clinical research suggests that the sole presence of APL antibodies rarely leads directly to thrombotic complications, only the induction of a general thrombotic stage is suspected, the clotting of which is subsequently triggered by infection or other effects such as pregnancy [4]. Thus, the etiopathology and clinical effects of APS remain a very complex medical condition, which will stay a relevant subject of research in the future.

In very rare cases, around less than 1%, there also appears a clinically more drastic variant of APS, the most common cause of which is an underlying infection: catastrophic anti-phospholipid syndrome (cAPS) [10].

Thus, the pending question to be answered in the context of the review concerns the prevalence of aPL antibodies in reported COVID-19 patients, in order to get an indication of their significance in COVID-19.

## Methods

To obtain the information used in this review, the PubMed Database was searched with the keywords “COVID-19” and “antiphospholipid syndrome” on 26/03/21; case reports and reviews were excluded beforehand from further analysis.

Of the studies found, only those addressing connections between APS and COVID-19, in particular the occurrence of APS typical antibodies in COVID-19, were included in further investigation.

In the following, the appearance of aPL antibodies and possible interactions in COVID-19 patients will be examined, and finally discussed in the “Conclusion” section.

## Results

For the initial search on PubMed, 114 search results were found in the mentioned period. In order to be shortlisted, the publications should investigate the prevalence of APS antibodies in a larger patient cohort and, at best, make statements on occurring thrombotic events. Accordingly, antiphospholipid antibody measurements from single patients were not mentioned here. These criteria were met by 18 publications at the end, the results of which are listed in Table 1. As an exception, the publication from Harzallah et al. was discovered through a published response, which was listed in the initial PubMed search. In addition, because of a recent review by Cavalli et al. [11], which raises the question of diagnostic and therapeutic perspectives of a secondary induced APS by COVID-19, two additional studies with important data on LAC prevalence, which were not included in the PubMed results, are mentioned.

**Table 1:** Summarized study results

Authors	Examined patients	Antibody prevalence	Thrombotic complications
Devreese et al.	31	16/31 LAC; 23/31 any aPL	9, thereof 7 positive for any aPL
Harzallah et al.	56	25/56 LAC; 5/56 IgG/IgM aCL or IgG/IgM anti-β2GPI	–
Gatto et al.	122	22.2% LAC; 13.4% IgG aCL; 2.7% IgM aCL, 6.3% IgG anti-β2GPI; 7.1% IgM anti-β2GPI	18/46
Galeano-Valle et al.	24	2/24 IgM aCL and IgM anti-β2GPI	24
Le Joncour et al.	104	35/104 aCL; 9/104 anti-β2GPI; 21/104 LAC; 49/104 any aPL	11; 64% positive for any aPL
Karahan et al.	31	8/31 any aPL; 6/26 LAC; 2/31 IgM aCL; 0/31 IgG/IgM anti-β2GPI; 2/31 IgA anti-β2GPI	–
Bowles et al.	34	31/34 LAC	–
Helms et al.	57	50/57 LAC	–
Borghi et al.	122	5.7/6.6% IgG/IgM aCL; 15.6/6.6/9.0%, IgG/IgA/IgM anti-β2GPI	16
Trahtemberg et al.	22	13/22 IgG aCL; 7/22 IgM aCL	–
Zuo et al.	172	52% any aPL	–
Amezcua-Guerra et al.	21	12/21 any aPL; 10%/14% IgG/IgM aCL	2
Hamadé et al.	41	7/41 any aPL; 5/41 LAC	9, thereof 2 positive for any aPL
Cristiano et al.	92	2/44 (early infection); 3/48 (late infection)	–
Reyes Gil et al.	68	30/68 LAC; 1/68 IgM aCL and IgM anti-β2GPI	19/30 (positive LAC)
Pineton de Chambrun et al.	25	92% LAC; 52% IgG/IgM aCL; 12% IgG/IgM anti-β2GPI	6, thereof all positive for any aPL
Xiao et al.	66 (critical condition); 13 (non-critical condition)	31/66 any aPL(IgG/IgM/IgA aCL and anti-β2GPI); 0/13 any aPL	–
Previtali et al.	35 (PM)	5/35 any aPL(IgG/IgA/IgM aCL and anti-β2GPI)	35

These publications have investigated the occurrence of APS-associated antibodies and have each come to different conclusions about their significance. Specifically, critical aPL antibodies such as LAC, anti-β2GPIb, and aCL were examined.

On the one hand, there were studies that found few positive aPL test results in their patient cohorts and were therefore critical of a pathogenic role of aPL antibodies in COVID-19. These included an examination of 31 patients with severe COVID-19 symptoms, who had to be treated at an ICU, in which antibodies were measured and then related to the occurrence of thrombosis [12]. The measured values did not suggest a direct connection of the aPL antibodies to thrombotic complications and lead to the assumption that the apparent LAC elevation occurred only intermittently because of the severe infection [12].

A similar result was obtained in a French publication concentrating mainly on LAC prevalence of the aPL antibodies, which tested 56 hospitalized patients. Again, a tendency for a transient increase in LAC due to primary COVID-19 infection was observed [13].

Furthermore, on LAC prevalence, one study in a group of 34 patients with prolonged activated partial thromboplastin time (aPTT) and COVID-19 infection found LAC in 31 patients, and consequently recommended further investigation of LAC in COVID-19-associated thrombotic events [14].

Another study also discovered detectable LAC levels in 50 out of 57 patients examined, but emphasized on the fact that the presence of LAC prior to the test cannot be ruled out [15].

A study based on the observation of 122 patients, whose measured aPL levels were subsequently compared with those of 157 primary anti-phospholipid syndrome (PAPS) patients and 91 other autoimmune rheumatic diseases (oARD), led to comparable conclusions [16]. To give special attention to the comparison with the studies already discussed, the LAC measurement resulted in a percentage of 22.2%, which is significantly higher in contrast to 14.6% in oARD, but does not show any comparison to the 64.1% in PAPS [16].

Finally, the prevalence of the measured antibodies was compared to the thrombosis occurring in 39.1% of 46 COVID-19 patients leading to no recognizable association, although it was not clear which specific group these 46 patients out of the initial 122 represent; aPL antibodies were therefore rejected as a useful indicator for thrombosis in COVID-19 patients [16].

A more critical judgement was reached after examination of 24 patients, who additionally had thrombotic complications during their treatment [17]. These patients were accordingly in an acute condition, but were not treated on an ICU. The following conclusion was in favor of denying the important role in the pathogenesis of VTE in severe cases of COVID-19 pneumonia by those aPL antibodies [17].

One particularly noteworthy study compared critically ill COVID-19 patients with non-COVID-19 critically ill patients and concluded that the prevalence of aPLs was about equal; only LAC showed an increased proportion in COVID-19 [18].

A similar result was obtained in a study focusing on the prevalence of thrombotic complications in a larger cohort of 122 patients, 16 of whom had severe thrombotic complications; anti-β2GPI IgG/IgA/IgM were at 15.6/6.6/9.0%, and aCL IgG/IgM at 5.7/6.6%; no relationship to thromboses could be observed [19].

In a further study, the course of APS antibodies of 20 critically ill patients without COVID-19 was compared with that of 22 critically ill COVID-19 patients. IgG aCL and IgM aCL were found most frequently with an increased occurrence of 13/22 and 7/22 in COVID-19 [20].

On the other hand, however, there was a study, the results of which suggest a greater influence of aPL than previously apparent; in a total of 172 COVID-19 patients undergoing treatment in hospital, a significantly high result of a positive detection of any of these antibodies in 52%, with varied stricter testing limits still in 30%, was observed indicating relevant involvement in the pathogenesis of COVID-19 [21].

Another study from Paris focused on non-ICU COVID-19 patients. In 47.1%, at least one of the aPL antibodies was detected. However, it was emphasized that persistency could not be excluded through repeated testing. It was concluded that aPL antibodies are common in moderate COVID-19 [22].

A case series study focused on the detection of aPL antibodies without LAC measurement and showed a high proportion with 57% [23].

Another study was able to detect a significant proportion of aPLs in ICU referrals with an aPL positive proportion of 7 out of 41 patients tested [24].

A study from New York retrospectively examined 187 patients tested for LAC within 2 months, only a part of whom had a positive COVID-19 test and found 30 increased LAC values in these 68 COVID-19-positive patients; interestingly, only 27 of 119 patients with negative COVID-19 test results were positive for LAC [25]. In addition, examination of the 30 patients with positive LAC and COVID-19 revealed 19 arterial or venous thrombotic events (63%), in notable contrast to 34% with LAC-negative results [25]. Subsequently, IgG/IgM aCL and anti-β2GPI antibodies were also tested in the LAC-positive patients, and IgM antibodies were found in only one patient [25]. Thus, in this study, it was concluded that there was a detectable association of elevated LAC levels in COVID-19 patients with thrombotic events [25].

Another study retrospectively evaluated 25 COVID-19-positive and ICU-treated patients, all suffering from acute respiratory distress syndrome (ARDS), for the presence of aPL antibodies. The results showed particularly high values for LAC with 92%, aCL with 52%, 12% anti-β2GPI, and 72% antiphospholipid antibodies [26]. Pulmonary embolism was observed in six patients, all of whom were also positive for all APL antibodies [26].

Another study found only a small number of detectable aPLs, but further raised the question of a “COVID-19-induced APS syndrome” due to increased non-criteria aPLs [27].

A further noticeable result originated from testing 66 patients in critical condition and 13 in non-critical condition for aPL antibodies [28]. The result showed that in the non-critical cases no elevated aPL antibodies were seen and in the 66 critical cases aPL antibodies were found in 31 [28]. This led to the assumption that despite the probably only transient increase caused by COVID-19, an APS-like syndrome could possibly be connected to COVID-19 [28].

Further investigation did explore the possibility of cAPS in COVID-19, using the clinical course and autopsies of 75 patients, who died of COVID-19 in Bergamo, as a basis [29]. Post-mortem, the serum of 35 patients, who were suspected of a possible cAPS, was tested. The results showed that the basic conditions of cAPS were indeed mostly fulfilled, including multi-organ involvement in a short time period and small vascular occlusions [29]. However, for cAPS to be confirmed, high levels of aPL antibodies would also have to be present—a condition that was not found to be existent; only in 5 cases could positive antibodies be detected, but these were only slightly above the threshold value [29]. Accordingly, cAPS involvement in pathogenesis was excluded.

## Discussion

Overall, the studies reviewed provide a concise general overview of the occurrence of aPL antibodies in COVID-19 patients showing a noticeably increased prevalence compared to a healthy population [11]. To come to a conclusion, the documented results will be compared in the following.

In total, 10 of the 18 publications analyzed cast doubt on a greater correlation between the formations of aPL antibodies in COVID-19 coagulopathy. Devreese et al. and Harzallah et al. showed a larger proportion of their patients with elevated LAC values, but few involvement in aCL and anti-β2GPI. Accordingly, both publications concluded that LAC levels alone may only be increased as a result of systematic viral infections, as the mechanism of forming of aCL and LAC in viral infections remains a matter of debate [30].

Gatto et al. also showed an increased LAC level, but of much greater importance, this study compared the antibody readings with existing rheumatic disease and especially with APS patients. This comparison showed that only few antibodies were comparable with these patient groups, and especially LAC in the COVID-19 cohort was far below that with APS. This shows that the occurrence of aPL antibodies, an important criterion for the diagnosis of APS, is therefore obviously much more drastic in APS than in COVID-19 patients indicating that the prevalence of acute COVID-19 with measurable aPL antibodies is not at all on the level of a distinctive clinical picture of an APS. Thus, LAC may be elevated due to the infection, but does not compare with APS.

A similar approach was taken by Karahan et al. who, after a comparison with non-COVID-19 critical patients, found only a slight increase in LAC, thus strengthening this assumption.

To also support these observations on the critical outcome side, the publication of Galeano-Valle et al., who did not test for LAC, again did not show a significant amount of elevated aPL antibodies, nor did Borghi et al.

Bowles et al. and Helms et al. showed relatively high values of positive LAC in their COVID-19 cohorts, although without further testing of other aPL antibodies for comparison.

On the other hand, however, 8 studies indicate that aPL antibodies can also take up a larger overall share in measurements, as shown for example in Zuo et al. and Reyes Gil et al. However, special attention is given to the observations of Pineton de Chambrun et al. and Xiao et al., whose publications show that the patient cohorts in these studies are related to patients in critical condition and treated on ICU. Thus, Pineton de Chambrun et al. indicate remarkably high values for aPL antibodies, especially for the almost ubiquitous LAC. And the comparison made by Xiao et al. also reveals a significantly higher proportion of aPL antibodies in COVID-19 patients in a more critical and acute condition.

In addition, it is also useful to put the thrombotic events in relation to the measured aPL antibodies. Unfortunately, not all publications provided clear figures on the incidence of thrombosis, which makes a precise analysis difficult. Pineton de Chambrun et al. and Reyes Gil et al. show that the majority of the detected thromboses also contained detectable aPL antibodies and a connection may be possible; but the results of Previtali et al. and Galeano-Valle et al. only found few antibodies despite many thromboses in their investigated group. In his publication, Devreese et al. also questioned the effect of aPL antibodies on thrombosis, although finding antibodies in most of them.

A recent study, of Trahtemberg et al., takes a special position, which is dedicated to a longitudinal observation of APS antibodies and compares these of COVID-19 ill patients with those of critically ill non-COVID-19 patients. The study showed that regardless of COVID-19, APS antibodies were detectable in many cases, but with a higher probability in COVID-19 patients [20]. Also striking was the fact that some of the patients, especially with COVID-19, already had aCL antibodies on admission [20]. Finally, the study also found a correlation between IgG aCL and general disease severity [20].

Another question to be answered concerns the close similarities in the pathogenesis of the COVID-19 and APS and, moreover, whether the COVID-19 infection could also induct cAPS. This question is based primarily on the fundamental changes in the COVID-19 coagulopathy already described above, and the induced hypercoagulable state in patients with COVID-19, suggesting the production of aPL antibodies and subsequent cAPS could be the cause of thrombosis as a previously unrecognized mechanism in critical disease states [31]. These assumptions of cAPS in COVID-19 could be challenged by the observations of Previtali et al. on the basis of a low level of detected aPL antibodies, but still at 6.7%, which is slightly higher than that in healthy patients. Thus, a role of cAPS in most of the 35 patients could be excluded by Previtali et al., but the percentage of positive aPL antibodies is nevertheless noticeable.

An important point also to be mentioned before the conclusion, regarding the assessment of a “COVID-19 induced APS,” concerns the necessity to remind here again of the criterion for APS. As already mentioned in the “Introduction,” according to the revised Sapporo classification, at least one clinical and one laboratory criterion are required [8]; thus, if transferred to the analyzed studies, only patients with the clinical criterion of vascular thrombosis can be considered. The laboratory criteria, on the other hand, require two successful measurements of LAC, aCL, or anti-β2GPI at an interval of 12 weeks, which was not performed in the analyzed studies. Also, the given titer limits, which are listed in the revised Sapporo classification, are only to be compared in isolated studies, so that also here no information is available on the basis of which an evaluation could take place.

Accordingly, the question of an APS syndrome induced by COVID-19 must clearly be discarded in the context of this review and can only be approached by follow-up studies that include consistent retesting of aPL antibodies. Only the question of an accumulation of aPL antibodies can be discussed properly.

Due to the apparent fact that LAC values are very frequently found in COVID-19 patients, it should be noted that they can also be distorted by false positive measurements, especially in the case of an increase in CRP, which is also to be expected in severe COVID-19 [32].

It is also problematic to reasonably interpret LAC values measured with concomitant anticoagulation therapy such as unfractionated heparin or vitamin K antagonists [33].

It is essential to further comment on the tested different antibodies that many of the studies described; i.e., IgAs were not always additionally included. Exact titers of the antibodies used as limits for positive results were not communicated in all cases. Thus, despite a wide range of studies, the direct comparability of the results is limited due to different test parameters and varying patient cohorts.

In order to summarize the previous findings before the conclusion, it is helpful to mention and include the results of other reviews in the same field.

Cavalli et al. who also examined APS and COVID-19 interestingly paid special attention to examining the prevalence of APS antibodies in a healthy population offering a comparison to COVID-19 patients, which showed overall higher values of aPL antibodies [11]. It called for further analysis of COVID-19 cohorts, which should be required to expand their observations to include measurement of aPL antibodies in relation to thrombosis and disease progression [11]. Also, a more detailed examination of the course of antibody presence was proclaimed to be performed for possibly confirming the disappearance or persistence of the antibodies after the acute phase [11].

Finally, further recent reviews that have addressed the same topic as this review, after assessing the published literature, have come to a similar conclusion to that reached here and from Cavalli et al. Pavoni et al. and Favaloro et al. elaborated in their conclusions on the limitations of the current data regarding a long-term existence of aPL antibodies [34, 35]. Likewise, Castillo-Martinez et al. discussed the question of an “epiphenomenon” [36] of the antibodies in contrast to a possible causality; as also mentioned here in the “Conclusion,” this review insists on longitudinal observations of the antibodies (with regard to the Revised Sapporo Classification), as it was implemented, e.g., by Trahtemberg et al. [36].

## Conclusion

In order to build on this discussion with the results found, the demand for more studies can only be urgently confirmed. A large-scale analysis of a COVID-19 cohort, whose antibody composition is put into context with the course of the disease and necessarily with thrombotic events, is essential [11]. In addition, the origin of aPL antibodies should also be given more attention: Some patients may already have APS before or APS may manifest after COVID-19, two questions that could not be discussed satisfactorily within the scope of this review. Subsequent studies with more frequent aPL testing and thus longitudinal observations in the course of the infection and most importantly at least 12 weeks afterwards may provide more answers. The results could also provide APS antibodies as a negative prognostic factor for a severe disease progression as Trahtemberg et al. have shown in their study.

The studies with the comparatively highest measurement results are noticeably more likely to be found in patients in a critical COVID condition. Consequently, the conclusions of many studies indicated an only transient increase of aPL antibodies due to COVID-19; the high proportion of positive LAC levels and discussions about aPL antibody formation after viral infections therefore lead to the need for additional investigation aimed at comparing the aPL antibody composition of LAC, aCL, and anti-β2GPI in COVID-19 with other viral infections.

Overall, most of the studies analyzed showed a significant proportion of detectable aPL antibodies, which was particularly noticeable in patients with severe disease progression and was much higher than the percentages of aPL antibodies in healthy subjects. On this basis, further clarifying studies are needed in order to explore the still unclear role of aPL antibodies and APS in COVID-19, thereby helping to better understand the pathomechanisms and pathogenesis of COVID-19.

## References

[1] Levi M, Thachil J, Iba T, Levy JH (2020). Coagulation abnormalities and thrombosis in patients with COVID-19. Lancet Haematol.

[2] Iba T, Levy JH, Connors JM, Warkentin TE, Thachil J, Levi M (2020). The unique characteristics of COVID-19 coagulopathy. Crit Care.

[3] Klok FA, Kruip M, van der Meer NJM, Arbous MS, Gommers D, Kant KM (2020). Incidence of thrombotic complications in critically ill ICU patients with COVID-19. Thromb Res.

[4] Radic M, Pattanaik D (2018). Cellular and molecular mechanisms of anti-phospholipid syndrome. Front Immunol.

[5] Erkan D, Lockshin MD (2010). Non-criteria manifestations of antiphospholipid syndrome. Lupus.

[6] Pericleous C, Ferreira I, Borghi O, Pregnolato F, McDonnell T, Garza-Garcia A, Driscoll P, Pierangeli S, Isenberg D, Ioannou Y, Giles I, Meroni PL, Rahman A (2016). Measuring IgA anti-beta2-glycoprotein I and IgG/IgA anti-domain I antibodies adds value to current serological assays for the antiphospholipid syndrome. PLoS One.

[7] Kungwankiattichai S, Nakkinkun Y, Owattanapanich W, Ruchutrakool T (2020). High incidence of antiphospholipid antibodies in newly diagnosed patients with lymphoma and a proposed aPL predictive score. Clin Appl Thromb Hemost.

[8] Miyakis S, Lockshin MD, Atsumi T, Branch DW, Brey RL, Cervera R (2006). International consensus statement on an update of the classification criteria for definite antiphospholipid syndrome (APS). J Thromb Haemost.

[9] Sene D, Piette JC, Cacoub P (2009). Antiphospholipid antibodies, antiphospholipid syndrome and viral infections. Rev Med Interne.

[10] Sciascia S, Lopez-Pedrera C, Roccatello D, Cuadrado MJ (2012). Catastrophic antiphospholipid syndrome (CAPS). Best Pract Res Clin Rheumatol.

[11] Cavalli E, Bramanti A, Ciurleo R, Tchorbanov AI, Giordano A, Fagone P, Belizna C, Bramanti P, Shoenfeld Y, Nicoletti F (2020). Entangling COVID-19 associated thrombosis into a secondary antiphospholipid antibody syndrome: diagnostic and therapeutic perspectives (Review). Int J Mol Med.

[12] Devreese KMJ, Linskens EA, Benoit D, and Peperstraete H. Antiphospholipid antibodies in patients with COVID-19: a relevant observation? J Thromb Haemost 2020. 10.1111/jth.14994 PMC7361253 32619328

[13] Harzallah I, Debliquis A, Drenou B. Lupus anticoagulant is frequent in patients with Covid-19. J Thromb Haemost 2020;18:2064–2065. 10.1111/jth.14867 PMC7264773 32324958

[14] Bowles L, Platton S, Yartey N, Dave M, Lee K, Hart DP, et al. Lupus anticoagulant and abnormal coagulation tests in patients with COVID-19. N Engl J Med. 2020;383:288–90. 10.1056/NEJMc2013656 PMC7217555 32369280

[15] Helms J, Tacquard C, Severac F, Leonard-Lorant I, Ohana M, Delabranche X (2020). High risk of thrombosis in patients with severe SARS-CoV-2 infection: a multicenter prospective cohort study. Intensive Care Med.

[16] Gatto M, Perricone C, Tonello M, Bistoni O, Cattelan AM, Bursi R (2020). Frequency and clinical correlates of antiphospholipid antibodies arising in patients with SARS-CoV-2 infection: findings from a multicentre study on 122 cases. Clin Exp Rheumatol.

[17] Galeano-Valle F, Oblitas CM, Ferreiro-Mazon MM, Alonso-Munoz J, Del Toro-Cervera J, di Natale M (2020). Antiphospholipid antibodies are not elevated in patients with severe COVID-19 pneumonia and venous thromboembolism. Thromb Res.

[18] Karahan S, Erol K, Yuksel RC, Artan C, Celik I. Antiphospholipid antibodies in COVID-19-associated pneumonia patients in intensive care unit. Mod Rheumatol. 2021:1–10. 10.1080/14397595.2021.1892257 PMC9383176 33620009

[19] Borghi MO, Beltagy A, Garrafa E, Curreli D, Cecchini G, Bodio C, et al. Prevalence, specificity, and clinical association of anti-phospholipid antibodies in COVID-19 patients: are the antibodies really guilty? medRxiv 2020.

[20] Trahtemberg U, Rottapel R, Dos Santos CC, Slutsky AS, Baker A, Fritzler MJ. Anticardiolipin and other antiphospholipid antibodies in critically ill COVID-19 positive and negative patients. Ann Rheum Dis. 2021:annrheumdis-2021-220206. 10.1136/annrheumdis-2021-220206 PMC8076626 33903092

[21] Zuo Y, Estes SK, Gandhi AA, Yalavarthi S, Ali RA, Shi H, et al. Prothrombotic antiphospholipid antibodies in COVID-19*.* medRxiv 2020.

[22] Le Joncour A, Frere C, Martin-Toutain I, Gougis P, Ghillani-Dalbin P, Maalouf G (2021). Antiphospholipid antibodies and thrombotic events in COVID-19 patients hospitalized in medicine ward. Autoimmun Rev.

[23] Amezcua-Guerra LM, Rojas-Velasco G, Brianza-Padilla M, Vazquez-Rangel A, Marquez-Velasco R, Baranda-Tovar F, et al. Presence of antiphospholipid antibodies in COVID-19: case series study*.* Ann Rheum Dis 2020. 10.1136/annrheumdis-2020-218100 32753426

[24] Hamade A, Woehl B, Harzallah I, Talbot M, Tousch J, Jambert L (2021). Antiphospholipid antibodies in patients with coronavirus disease 2019 infection hospitalized in conventional unit. Blood Coagul Fibrinolysis.

[25] Reyes Gil M, Barouqa M, Szymanski J, Gonzalez-Lugo JD, Rahman S, Billett HH (2020). Assessment of lupus anticoagulant positivity in patients with coronavirus disease 2019 (COVID-19). JAMA Netw Open.

[26] Pineton de Chambrun M, Frere C, Miyara M, Amoura Z, Martin-Toutain I, Mathian A, et al. High frequency of antiphospholipid antibodies in critically ill COVID-19 patients: a link with hypercoagulability? J Intern Med 2020. 10.1111/joim.13126 PMC7307032 32529774

[27] Cristiano A, Fortunati V, Cherubini F, Bernardini S, Nuccetelli M (2021). Anti-phospholipids antibodies and immune complexes in COVID-19 patients: a putative role in disease course for anti-annexin-V antibodies. Clin Rheumatol.

[28] Xiao M, Zhang Y, Zhang S, Qin X, Xia P, Cao W, Jiang W, Chen H, Ding X, Zhao H, Zhang H, Wang C, Zhao J, Sun X, Tian R, Wu W, Wu D, Ma J, Chen Y, Zhang D, Xie J, Yan X, Zhou X, Liu Z, Wang J, du B, Qin Y, Gao P, Lu M, Hou X, Wu X, Zhu H, Xu Y, Zhang W, Li T, Zhang F, Zhao Y, Li Y, Zhang S (2020). Antiphospholipid antibodies in critically ill patients with COVID-19. Arthritis Rheumatol.

[29] Previtali G, Seghezzi M, Moioli V, Sonzogni A, Cerutti L, Marozzi R, Ravasio R, Gianatti A, Guerra G, Alessio MG (2020). The pathogenesis of thromboembolic disease in COVID-19 patients: could be a catastrophic antiphospholipid syndrome?. Thromb Res.

[30] Uthman IW, Gharavi AE (2002). Viral infections and antiphospholipid antibodies. Semin Arthritis Rheum.

[31] Mendoza-Pinto C, Escarcega RO, Garcia-Carrasco M, Bailey DJO, Galvez-Romero JL, Cervera R (2020). Viral infections and their relationship with catastrophic antiphospholipid syndrome: a possible pathogenic mechanism of severe COVID-19 thrombotic complications. J Intern Med.

[32] Connell NT, Battinelli EM, Connors JM. Coagulopathy of COVID-19 and antiphospholipid antibodies. J Thromb Haemost. 2020. 10.1111/jth.14893 PMC7267637 32379918

[33] Pengo V, Tripodi A, Reber G, Rand JH, Ortel TL, Galli M, de Groot PG, Subcommittee on Lupus Anticoagulant/Antiphospholipid Antibody of the Scientific and Standardisation Committee of the International Society on Thrombosis and Haemostasis (2009). Update of the guidelines for lupus anticoagulant detection. Subcommittee on Lupus Anticoagulant/Antiphospholipid Antibody of the Scientific and Standardisation Committee of the International Society on Thrombosis and Haemostasis. J Thromb Haemost.

[34] Favaloro EJ, Henry BM, and Lippi G. COVID-19 and antiphospholipid antibodies: time for a reality check? Semin Thromb Hemost 2021. 10.1055/s-0041-1728832 34130340

[35] Pavoni V, Gianesello L, and Horton A. Antiphospholipid antibodies in critically ill COVID-19 patients with thromboembolism: cause of disease or epiphenomenon? J Thromb Thrombolysis 2021. 10.1007/s11239-021-02470-y PMC8109223 33973157

[36] Castillo-Martinez D, Torres Z, Amezcua-Guerra LM, Pineda C (2021). Are antiphospholipid antibodies just a common epiphenomenon or are they causative of immune-mediated coagulopathy in COVID-19?. Clin Rheumatol.

